# Association between dietary self-perception and sugar-sweetened beverage consumption among young adult Latinas

**DOI:** 10.1017/S1368980026102523

**Published:** 2026-04-10

**Authors:** Mi Zhou, A Susana Ramírez, Xinyu Xu, Jacqueline Bergman

**Affiliations:** 1 Department of Nutrition, Food Science, and Packaging, San Jose State University, USA; 2 Department of Public Health, University of California Merced, USA

**Keywords:** Sugar-sweetened beverages, Dietary self-perception, Latinas, Acculturation, Beverage consumption, Dietary behaviours

## Abstract

**Objective:**

To investigate the relationship between self-perceived overall dietary healthfulness and self-reported sugar-sweetened beverage (SSB) consumption among young adult Latinas, accounting for socio-economic and acculturation-related factors.

**Design:**

Cross-sectional analysis using survey data. SSB intake was assessed using the BEVQ-15, and dietary self-perception was measured via a two-item scale. Multiple linear regression models examined associations between self-perception and total daily SSB intake, adjusting for income, education and two validated acculturation indicators.

**Setting:**

Participants were recruited from a national online panel across the USA.

**Participants:**

A total of 881 Latina women aged 18–29 years participated. After removing cases with invalid outcome responses and outliers, 840 and 829 were included in descriptive and regression analyses.

**Results:**

Better dietary self-perception was significantly associated with greater total SSB intake in both unadjusted (B = 1·74, P = 0·048) and fully adjusted models (B = 2·10, P = 0·017). Lower income (B = –0·64, P = 0·031) and lower education (B = –0·77, P = 0·026) were also associated with higher intake. Acculturation variables were NS. Subcategory models showed positive associations between self-perception and sweet tea (B = 0·99, P < 0·001) and black coffee/tea with sugar (B = 0·51, P < 0·01) and a marginal inverse association with soft drinks (B = –0·47, P = 0·060).

**Conclusions:**

Young Latinas who perceive their diets as healthy may consume more added sugar from beverages than recommended. Public health efforts should address this perception gap and emphasise culturally relevant messaging about hidden sugars in commonly consumed drinks.

Excessive consumption of sugar-sweetened beverages (SSB), including soft drinks, sweetened teas and flavored coffees, is a well-documented public health concern due to its strong association with obesity, type 2 diabetes, CVD and dental caries^(1–3)^. Despite broad public health efforts to limit SSB intake, consumption remains disproportionately high among young adults and certain racial/ethnic minority populations in the USA, particularly Latina women^(4–8)^. These disparities contribute to higher rates of obesity and related chronic conditions compared with non-Hispanic Whites^(9)^.

Dietary behaviours are shaped by individual perceptions, social and cultural factors and environmental influences^(10,11)^. Within this context, self-perceived dietary healthfulness emerges as a crucial individual-level factor that could influence dietary behaviours. Consistent with prior literature, dietary self-perception here refers to individuals’ subjective assessment of the overall healthfulness of their eating patterns, distinct from constructs such as dietary knowledge or self-efficacy. Some research suggests that higher self-perceived diet quality is associated with more optimal nutrient intake^(12–14)^. However, other research shows that individuals’ perceptions of their diet quality do not always align with objective measures of nutritional intake. For instance, Sharif and colleagues observed that daily consumption of energy-dense foods including Mexican pastries, chips, crackers and ice cream remained high among participants regardless of whether they rated their eating habits as ‘good’ or ‘poor’ among Latinos^(13)^. A study using NHANES data found that perceived diet quality was associated with higher measured diet quality among White and Black individuals, but not Mexican Americans^(15)^. These discrepancies from the previous research, particularly among Latinos, underscore the complexity of the relationship between self-perceived diet quality and measured nutritional intake and highlight a notable gap in understanding the factors that drive SSB intake among Latinos.

In addition to self-perceptions of dietary quality, socio-economic status (SES), particularly education and income, is typically associated with healthier dietary choices and nutritional literacy^(16–18)^. Acculturation, defined as the process through which individuals adopt cultural norms and behaviours of a dominant society, also plays a pivotal role in dietary behaviours among immigrant populations^(19,20)^. For Latinas in the USA, higher levels of acculturation have frequently been associated with increased consumption of processed and convenience foods, including SSB^(21–23)^. Nonetheless, the relationship between acculturation and dietary behaviours remains complex and warrants further examination, particularly in younger cohorts^(24–26)^.

Despite recognition of these interrelated factors, existing research often overlooks how individual dietary self-perceptions intersect with SES and acculturation among Latina young adults, especially regarding specific dietary behaviors like SSB consumption. Understanding these relationships is critical for designing effective public health interventions and targeted nutrition education programmes that align more closely with this group’s perceptions and lived experiences. Therefore, the primary objective of this study is to examine the association between overall dietary quality self-perception and self-reported SSB consumption among young adult Latinas, accounting for socio-demographic and cultural factors including education, income and acculturation.

## Methods

### Study design and sampling

This study employed a cross-sectional survey design. Participants were recruited in 2023 through Ipsos, a commercial research panel provider with a diverse, nationally representative pool. Eligible participants identified as female, aged 18–29 years, Latina or Hispanic and currently residing in the USA. Additional inclusion criteria required English proficiency to align with the broader project’s focus on acculturation and health behaviour among more-acculturated Latinas. The study protocol was approved by the Institutional Review Board of University of California, Merced (ID: UCM15-0017). Participants received compensation in accordance with Ipsos’s panel policies.

### Measures

#### Sugar-sweetened beverage consumption

SSB intake was measured using the validated BEVQ-15^(27)^, which assesses frequency and volume of consumption for various beverage types over the past month. Monthly beverage intake was converted to mean daily consumption using standardised BEVQ-15 scoring procedures. Total daily SSB consumption was computed by aggregating responses for: (1) regular soft drinks; (2) sweetened juice beverage/drink (e.g. fruit punch); (3) regular energy and sports drinks (e.g. Red Bull); (4) Sweet tea (e.g. Lipton sweet tea); (5) black coffee or tea with sugar added and (6) coffee or tea with milk and/or creamer with sugar added.

#### Dietary self-perception

Self-perceptions of dietary health were two items adapted from the U.S. Food and Drug Administration’s ratings of self-perceived healthiness of dietary behaviours (Cronbach alpha = 0·63)^(28)^. One question asked participants to reflect on their eating habits and rank the overall healthiness of their diet; responses ranged from 1 (poor) to 5 (excellent). The other question asked them to indicate if they are confident in their ability to choose healthy foods; responses ranged from 1 (strongly disagree) to 5 (strongly agree).

#### Acculturation

Acculturation was assessed using two validated scales capturing complementary dimensions of acculturation. The Acculturation, Habits, and Interests Multicultural Scale for Adolescents^(29)^ scale includes eight items (Cronbach alpha = 0·79), addressing various aspects such as the types of people participants are comfortable around or feel the best fit, the countries of origin for their best friends, favourite music and TV shows, holidays they celebrate, the cuisine they consume at home and the influence of their country of residence on their way of thinking and doing things. Response options range from 1 (neither US nor the country they’re from) to 4 (US). Higher scores indicate greater acculturation to US culture. We also used the Short Acculturation Scale for Hispanics^(30)^ (Cronbach alpha = 0·89) to assess linguistic acculturation across five domains: their general spoken language, reading language, language spoken at home, language of internal thought and language used when conversing with friends. Each item was rated from 1 (‘Spanish only’) to 5 (‘English only’), with higher values indicating greater acculturation to English-speaking culture.

#### Socio-demographic covariates

Participants reported their age (in years), highest level of education (on a 1–13 scale, with 13 indicating a doctoral degree) and annual household income (on a 1–9 scale, with 9 indicating more than $100 000).

### Analytic approach

Item responses were averaged to generate composite scores for dietary self-perception and acculturation measures. Education and income were analysed using their original ordinal scale values, with Table 1 categories presented descriptively only. Age was not included due to the narrow sample age range (18–29 years).

**Table 1.** Sample characteristics (*n* 840 Latina women)

Variable	Total (*n* 840)	%	Mean	sd
Age (years)				
Range (18–29)	840		23	3·24
Education				
Below college	352	41·9 %		
College or above	488	58·1 %		
Household income				
Below $50 000	598	71·2 %		
Above $50 000	242	28·8 %		
Acculturation level				
Habits (1–4)			3·16	0·42
Language (1–5)	840		3·77	0·80
Self-rated healthiness of diets				
Range (1–5)	840		3·40	0·82

Note: SD = standard deviation.

Descriptive statistics were calculated for all study variables. Multiple linear regression models were estimated to examine the association between dietary self-perception and daily SSB consumption. Model 1 included only dietary self-perception as a predictor. Model 2 adjusted for education and income. Model 3 additionally included acculturation score. Model diagnostics were conducted to assess multicollinearity, normality of residuals and influential outliers. All analyses were conducted in SPSS Version 29. Statistical significance was evaluated at an alpha level of 0·05.

## Results

A total of 881 participants completed the survey. We removed participants who reported not knowing their family income, resulting in a final sample of 840 participants (Table 1). The mean age of participants was 23 years (sd = 3·2). More than two-thirds (71·2 %) reported annual household incomes below $50 000 and 41·9 % had not completed a college degree. The mean score for dietary self-perception was 3·40 (sd = 0·82) on a five-point scale.

Regarding SSB intake, the mean total daily SSB intake was 643 ml (sd = 721), substantially exceeding the 355 ml recommended daily limit by the American Heart Association^(31)^. Overall, 7·3 % reported no SSB consumption, 39·3 % reported consumption within the recommended daily limit (≤ 355 ml) and 53·5 % exceeded the recommended limit. Among beverage categories, regular soft drinks and sweet tea were the most frequently consumed, averaging 166 ml and 118 ml per day, respectively.

As shown in Table 2, in the unadjusted model (Model 1), higher self-perception was significantly associated with greater daily SSB intake (B = 1·74, SE = 0·88, *P* < 0·05). This association remained significant after adjusting for income and education (Model 2) and in the fully adjusted model including acculturation variables (Model 3, B = 2·10, se = 0·88, *P* < 0·05). Income and education were both negatively associated with SSB consumption in adjusted models. Acculturation by habits and language were not significantly associated with intake in the final model.

**Table 2.** Regression models for daily SSB intake: unadjusted, sociodemographic-adjusted and fully adjusted (outlier removed, *n* 829)

	Model 1: Unadjusted		Model 2: +Income & Education		Model 3: +Acculturation	
Predictor	B	se	B	se	B	se
Self-perception	1·737^*^	0·876	2·080^*^	0·875	2·099^*^	0·875
Income	–		−0·649^*^	0·295	−0·641^*^	0·297
Education	–		−0·722^*^	0·341	−0·766^*^	0·343
Acculturation (habits)	–		–		2·096	1·875
Acculturation (language)	–		–		−1·156	1·002

Note: Values are unstandardised regression coefficients (B) with standard errors (SE).

Dependent variable: Daily overall sugar-sweetened beverage (SSB) intake.

* *P* < 0·05.

In fully adjusted models for SSB subcategories (Table 3), self-perception was positively associated with sweet tea (B = 0·99, *P* < 0·001) and black coffee/tea with sugar added (B = 0·51, *P* < 0·01) and marginally associated with lower soft drink consumption (B = –0·47, *P* = 0·060). No significant associations were observed for sweetened juice, sports drinks or coffee/tea with creamer. Education was inversely associated with soft drink and sweet tea consumption, while income was negatively associated with sweet tea only. Acculturation variables showed no consistent associations; acculturation by habits approached significance for coffee/tea with creamer (B = 0·72, *P* = 0·062).

**Table 3.** Multivariable linear regression results for predictors of daily intake across SSB subcategories (outlier removed, *n* 829)

	Self-perception		Income		Education		Acculturation-habits		Acculturation-language	
SSB subcategory	B	*P*-value	B	*P*-value	B	*P*-value	B	*P*-value	B	*P*-value
Sweetened juice	0·146	0·372	−0·054	0·331	−0·109	0·086	−0·117	0·736	−0·206	0·269
Soft drink	−0·466	0·060	0·031	0·712	−0·228	0·018*	0·278	0·598	−0·229	0·417
Sports drink	0·362	0·068	0·010	0·879	−0·095	0·215	0·302	0·475	−0·190	0·402
Sweetened tea	0·987	0·000***	−0·184	0·002**	−0·181	0·018*	−0·148	0·730	−0·068	0·763
Black coffee/Tea with sugar added	0·507	0·001**	−0·079	0·129	−0·054	0·371	−0·168	0·610	−0·244	0·166
Coffee/Tea with milk/Creamer with sugar added	0·327	0·071	−0·024	0·691	−0·011	0·882	0·724	0·062	−0·121	0·558

Note: Values are unstandardised regression (B) coefficients with *p*-values.

Dependent variable: Daily sugar-sweetened beverage (SSB) intake across beverage sub-categories.

*P < 0·05, **P < 0·01, ***P < 0·001.

## Discussion

This study examined the relationship between young adult Latinas’ self-perceived dietary healthfulness and their self-reported SSB consumption, accounting for socio-economic and acculturation-related factors. Contrary to expectations, participants who rated their diets as healthier reported significantly *greater* daily SSB intake than those with less-healthy diets, even after adjusting for education, income and acculturation. This finding highlights a potential misalignment between individuals’ perceptions or understanding of their dietary quality and their self-reported consumption in this population. These results add nuance to previous studies suggesting that positive dietary self-perception is typically associated with healthier behaviours^(12–14)^. However, the cross-sectional design prevents determining directionality; individuals with higher SSB intake may self-justify their dietary self-perception to reduce the discomfort of conflicting beliefs and behaviours^(32)^.

SSB consumption patterns are not uniform across beverage types; soft drink consumption was lower among individuals with higher education and those who reported better-quality diets, reflecting the impact of long-standing public health messaging about the harms of soda. However, higher self-perception was linked to greater intake of sweet tea drinks and coffee/tea with added sugar, suggesting these drinks may be viewed as healthier or more acceptable than sodas. These beverages may also carry cultural familiarity or positive cultural associations that make them seem more acceptable or less ‘unhealthy’ than soda, contributing to a disconnect between their perceived and nutritional healthfulness^(33)^. Public health efforts must move beyond a soda-centric approach and begin addressing sugar-containing beverages that are often perceived as healthy, such as sweetened tea, juice and specialty coffee drinks, which were frequently consumed in this sample.

Our findings also reaffirm known socio-economic gradients in dietary behaviour. Participants with higher income and education levels consumed significantly less SSB. These disparities are well-documented in public health literature and likely reflect a combination of greater health literacy, access to healthier beverage options and reduced exposure to targeted SSB marketing. Notably, neither of the acculturation measures was significantly associated with SSB intake, suggesting that among this relatively acculturated sample of English-speaking young Latinas, variation in cultural orientation may exert less influence on beverage choices than other structural and perceptual factors. Further research is warranted to examine whether different dimensions or degrees of acculturation affect SSB consumption in more diverse or less acculturated Latino populations.

Most participants consumed SSB above recommended limits, underscoring the need to address excessive intake among young adult Latinas. This highlights the need for culturally tailored strategies promoting healthier beverage choices.

Limitations of this study include the cross-sectional design, which precludes causal inference, and reliance on self-reported intake data, which may be subject to recall or social desirability bias. Because the sample was drawn from an English-speaking online panel, it likely reflects a more acculturated subset, limiting generalisability and possibly contributing to the null acculturation findings. Also, the Spanish-based acculturation scales limit generalisability to Latinas from non-Spanish-speaking backgrounds, such as Portuguese-speaking Brazilians or Indigenous. Furthermore, we did not assess chronic disease history, dietary restrictions or other socio-economic, behavioural or environmental influences, which could affect dietary behaviours and may limit interpretation of the findings. Finally, the two-item measure of dietary self-perception demonstrated modest internal consistency and reflects only limited aspects of the broader construct of perceived dietary healthfulness, which should be considered when interpreting the findings.

In conclusion, these findings suggest that interventions to reduce SSB consumption among young adult Latinas should increase awareness of added sugars in culturally normative beverages and encourage critical reflection on the definition of a ‘healthy diet.’ Addressing culturally familiar drinks, such as sweetened teas and sweetened coffee beverages, may be particularly important. These findings also suggest that SSB taxation policies may be more effective when paired with communication strategies clarifying the sugar content of beverages often perceived as healthier.
